# 25 years of telepathology research: a bibliometric analysis

**DOI:** 10.1186/1746-1596-6-S1-S26

**Published:** 2011-03-30

**Authors:** Vincenzo Della Mea

**Affiliations:** 1 University of Udine, Medical Informatics, Telemedicine and eHealth Lab, Dept. of Mathematics and Computer Science, Italy

## Abstract

**Background:**

The first appearance of the word “telepathology” in a scientific paper can be tracked down to 1986, in a famous editorial of Ronald Weinstein. Since that paper, research in telepathology grew up developing different subfields, including static and dynamic telepathology and more recently virtual microscopy. The present work attempts an analysis of research in telepathology, starting from the tools provided by bibliometrics.

**Methods:**

A query has been developed to extract papers related to telepathology and virtual microscopy, and it has been then submitted to Pubmed by means of Entrez Utilities functions. Results obtained in XML have been processed through ad-hoc developed PHP scripts, in order to extract data on Authors, countries, and keywords.

**Results:**

On PubMed, 967 papers related to telepathology and virtual microscopy have been retrieved, which involved 2904 Authors; corresponding authors were from 37 countries. Of those authors, 2213 co-authored just one paper. Papers were published on 344 different journals, of which only 52 from the Pathology field. An analysis of papers per year has been also attempted, that demonstrates variable research output in time.

**Conclusions:**

From the proposed analysis, telepathology seems to have been consistently studied, in time, by about 400 researchers, with occasional participation of many other people. Telepathology research seems also to have varied in time, although some peaks in paper publishing are certainly related to the proceedings of the European congress on telepathology series, when they have been published on journals. However, some clear sign appears that suggests research in traditional telepathology, after a peak in 2000, showed some decline until virtual microscopy became mainstream, topic that currently pushes research again. The low number of clinical trials calls for more randomized studies in telepathology, to enable evidence-based application.

## Background

The first appearance of the word “telepathology” in a scientific paper can be tracked down to 1986, in a famous editorial of Ronald Weinstein [1]; in 1996 it was then inserted into the MeSH term list. Since that paper, research in telepathology grew up developing different subfields, including static and dynamic telepathology , and more recently also virtual microscopy.

Telepathology, as also related research topics as pathology image analysis and pathology-related information systems, represents a niche area of research in the wider pathology field, with contributions coming from medical informatics and biomedical engineering areas, in a interdisciplinary way. It is also a subspecialty of the larger field of telemedicine, where more historical fields include teleradiology and telecardiology.

Bibliometrics utilizes quantitative analysis and statistics to describe patterns of publication within a given field or body of literature. It is becoming a tool for research evaluation and management, through the use of quantitative bibliometric indicators in the processes of research funding, academic promotion, and recruitment, although many criticisms on unwanted consequences have been made [3]. Bibliometrics intersects with scientometrics, which is aimed at science (and research) study and evaluation by means of any of the products of the scientific process, and not only basing on literature.

Another application of bibliometrics is the analysis of research trends, offering insights on research developments in a specific field over time, by assuming that publications represent, at a specific time, the output of research efforts in the immediately preceding years.

Bibliometric analysis has been rarely applied to pathology research, although some report exists on the overall scientifi production [4] and on specific areas [5,6]. Also telemedicine has been the subject of bibliometric analyses in few studies [7-9].

After 25 years from the appearance of telepathology in scientific literature, the present work attempts an analysis of research in this field, starting from the techniques provided by bibliometrics.

## Methods

### Source of data and software

Data of scientific articles related to telepathology have been extracted from the electronic database PubMed [10], by means of semiautomated procedures.

A generic tool (MedMine) has been developed in PHP for submitting queries to Pubmed by means of Entrez Programming Utilities functions [11]. The results obtained in XML format have been then processed by the tool to extract data on Authors, countries, keywords and journals.

A query has been then developed to extract papers related to telepathology and virtual microscopy, and provided as input to the MedMine software.

Results were saved in CSV format and then graphed using Excel (Microsoft, Redmond, USA). Geographical map has been drawn using Geocommons [12].

### Search strategy

The term “telepathology” has been included into the MeSH terminology since 1996, but however it was already in use in scholarly publication. In addition to that, the term is not sufficient to trace all papers whose content is related to telepathology, since sometimes “telecytology” has been used in the past. The later evolution of telepathology towards virtual microscopy (also called digital microscopy or, more recently, Whole Slide Imaging) has been registered even into a change in the name of the European Congress on Telepathology, which 8 years ago added “and Virtual Microscopy” to its name. All these terms are related to telepathology research and thus have to be comprised in a query aimed at retrieving papers on telepathology.

Thus, the query developed to retrieve as much as possible papers related to telepathology techniques has been defined as follows, after some attempts:

"telepathology"[MeSH Terms] OR "telepathology"[All Fields]

OR "telecytology"[All Fields]

OR "virtual microscopy"[All Fields] OR "virtual microscope"[All Fields]

OR "digital slides"[All Fields] OR "digital microscopy"[All Fields]

OR "digital slide"[All Fields]

*OR* (*"whole slide"[All fields] AND*

(*“imaging”[All Fields] OR “images”[All Fields]*

*OR “scanning”[All Fields]*))

The term “whole slide imaging” is not comprised in the query because, at the time of work, PubMed did not yet index any article with that term (source: PubMed helpdesk), although it is used in some papers.

Two slightly revised versions and mutually exclusive of the query have been created to retrieve traditional telepathology papers and virtual or digital microscopy papers.

In addition to that, to identify journal categories, three queries have been made on PubMed on the Journals database, to extract pathology, medical informatics and biomedical engineering journals according to categories coded in the Journals database.

### Collected data

Article data obtained from PubMed were used to extract Authors, journal, publication date and type, MeSH keywords, and country of the corresponding Author when available.

From such data, further processing was done to identify the distribution of papers in time, by authors, by country, by journal, and also keyword frequency.

Additional data included the number of Authors per paper, and the journal topic when among Pathology, Medical Informatics or Biomedical Engineering.

## Results and discussion

**Figure 1:**
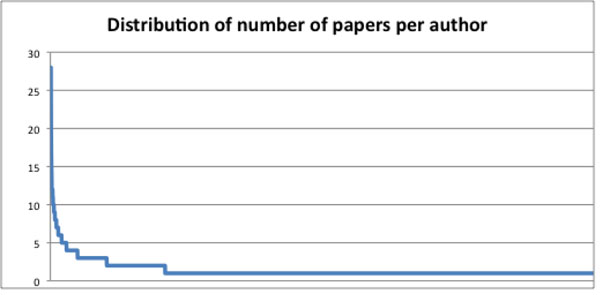
Distribution of number of papers per author

On PubMed, 967 papers related to telepathology and virtual microscopy have been retrieved, which involved 2904 Authors. Of those authors, 2213 co-authored just one paper, so they can be considered occasional telepathology authors; 312 co-authored two papers. Figure 1 shows the distribution of papers per Author, that demonstrate the classical “long tail” aspect of many network-related processes. The core community of telepathology researchers can be thus circumscribed under 400 scientists.

The average number of authors per paper was 4.47.

**Figure 2:**
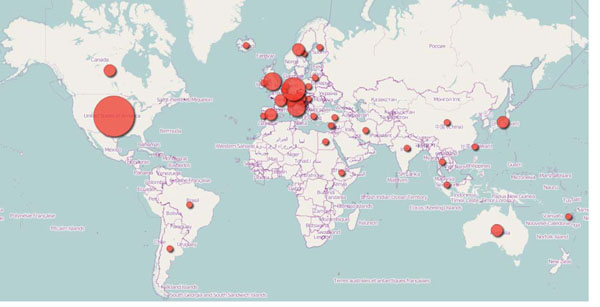
**Number of papers per country**. Size of circles is correlated to number of papers published.

Corresponding Authors, as recorded in the Affiliation field of PubMed data, were from 37 different countries. However, for 217 papers (22.4%) it was not possible to identify country of affiliation for a number of reasons, and in particular some paper did not report affiliation at all while some paper, specially in national journals, did not have the country in the Author address. Figure 2 outlines country contributions, then described in table 1.

**Table 1:** Papers per country

country	papers
United states of America	310
Germany	81
Italy	46
United Kingdom	40
Japan	27
France	23
Australia	21
Canada	18
Austria	17
Switzerland	17
Norway	16
Spain	14
Poland	13
Hungary	13
Sweden	12
Netherlands	10
China	9
Ireland	9
Finland	8
India	6
Croatia	6
Taiwan	5
Greece	4
Belgium	4
Iran	3
Thailand	3
Brazil	3
Lithuania	2
Cyprus	2
Turkey	1
Portugal	1
Fiji	1
Argentina	1
Egypt	1
Singapore	1
Ethiopia	1
Iceland	1

Papers were published on 344 different journals, of which only 52 from the Pathology field (accounting for 372 papers), 24 from Medical Informatics field (163 papers), and 8 from Biomedical Engineering (18 papers). In the period examined, a total of 171 journals has been active in the Pathology field, so that 30.4% of them published telepathology papers.

Other journals include some generalist publication, and many journals of fields that can be interested in telepathology results, including oncology, dermatology, ophtalmology. Table 2 shows journals that hosted more than ten telepathology papers.

**Table 2:** Major telepathology-publishing journals

Journal	n. of papers	Category
J Telemed Telecare	71	Medical Informatics
Hum Pathol	65	Pathology
Telemed J	27	Medical Informatics
Stud Health Technol Inform	24	Medical Informatics
Diagn Pathol	23	Pathology
Anal Cell Pathol	23	Pathology
Adv Clin Path	22	Pathology
Arch Anat Cytol Pathol	22	Pathology
Arch Pathol Lab Med	18	Pathology
Am J Clin Pathol	17	Pathology
Pathologe	16	Pathology
J Clin Pathol	15	Pathology
Histopathology	11	Pathology
Tidsskr Nor Laegeforen	10	General medicine
Zentralbl Pathol	10	Pathology

Only 18 papers (1.9%) were related to clinical trials as stated in the publication record by the publication type field.

**Figure 3:**
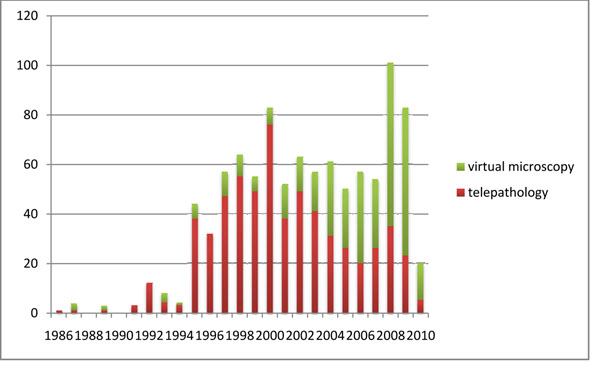
Number of papers per year

An analysis of papers per year has been also attempted, that demonstrates variable research output in time, as can be seen in figure 3. This variability may have different justifications, however at least two are major reasons. One is the bi-annual European Congress of Telepathology, which proceedings have been often published in special issues of journals, thus giving a cycling aspect to scientific productivity. The other is the birth and diffusion of virtual microscopy techniques, which by the way might be traced back to 1997 [13,14], although at least a paper that used a basic version of matrix-based acquisition for image analysis appeared in 1995 [15]. Thus, papers referring to digital microscopy term before 1997 are in fact not related to what is currently called in that way (although they have been left in the chart). Virtual microscopy research overcome traditional telepathology in 2006, according to number of published papers.

Finally, also MeSH keyword used to index articles have been examined to understand the topics dealt with by articles. From data presented in Table 3 (where only keywords with more than 50 occurrences have been listed), telepathology has been applied mostly to human pathology, although a number of papers dealt with veterinary applications. Technical topics strongly related to telepathology are image processing, software, interfaces, and networks. The two main applications seems to be remote consultation and frozen sections. There has been many studies related to the quality of the process, expressed in terms of reproducibility, sensitivity and specificity, and interobserver variation.

**Table 3:** Main MeSH keywords for telepathology papers

Keyword	Occurrences
Humans	732
Telepathology	528
Microscopy	186
Image Processing, Computer-Assisted	178
Female	154
Internet	142
Remote Consultation	120
Male	114
Telemedicine	97
Reproducibility of Results	93
Pathology, Clinical	92
User-Computer Interface	87
Software	84
Computer Communication Networks	78
Animals	72
Pathology	71
Adult	65
Middle Aged	64
Frozen Sections	56
Sensitivity and Specificity	53
Aged	53
Observer Variation	52
Neoplasms	51

### Limits of this study

The query used might have retrieved also some papers related to image analysis, due to the sometimes wide meaning given to the terms included in the query. On the other side, researchers in telepathology field almost always had a similar commitment to other computer-based techniques, including image processing and analysis.

As already noted, some article did not present complete affiliation data, so a fair number of articles were not assigned to a country. This might have brought to an underestimation for many countries, but hopefully evenly distributed, thus we did not manually correct data, following the same approach as in [7]. The method of using corresponding Author address for identifying country of origin, although often used, also does not allow to recognize transnational research.

Some surnames were written with variants, including variable transliteration of diacritic characters, or typographical errors, or wrong abbreviation of multiple surnames. Thus individual contribution of some Authors is underestimated, although some correction has been done by hand for recovering as much data as possible.

No evaluation of citation data has been attempted, because the aim of the work was to describe research efforts more than impact.

## Conclusions

From the proposed analysis, telepathology seems to have been studied, in time, by a relatively small core community of about 400 researchers, with occasional participation of many other people. If, from one side, this may seem to circumscribe research to a small number of passionates, on the other side the involvement of about 2500 occasional co-authors might be interpreted as a good dissemination activity towards physicians involved in clinical practice and/or other research.

The most prolific Authors have been, unsurprisingly, the two “fathers” of telepathology: Ronald Weinstein and Klaus Kayser. They contributed with 28 papers each to the birth and growth of telepathology, and with other papers to other image-related techniques.

Interestingly, while analysing the literature, the term “digital microscopy” have been found in the title of a 1984 paper by Bartels et al. [16], where it was used to identify the various applications of microscopy digital images, so before the rise of telepathology and virtual microscopy.

USA contributes with just less than one third of the total number of published papers; summed up, papers from European countries reach a similar amount. Although the number of involved countries seems low, it is in line with the 42 countries involved in telemedicine up to 2003 [7], and with the 25 countries having published on the Journal of Telemedicine and Telecare [17]. Telemedicine research has been reported to be correlated with human development index, GNP per capita, and number of PCs per 1000 inhabitants [7]. The same paper reports that also a low population density is slightly correlated with telemedicine studies, although in a not significant way.

In respect to its parenty specialty telemedicine, according to [7] telepathology accounts for 5.3% of papers.

From the analysis of hosting journals, it clearly appears that telepathology is a multidisplinary area, with input from Pathology but also from the Medical Informatics field, and with interested parties all along the path served by Pathology specialists.

Telepathology research seems also to have varied in time, although some peaks in paper publishing are certainly related to the proceedings of the european congress on telepathology series, when they have been published on journals. However, some clear sign appears that suggests research in traditional telepathology, after a peak in 2000, showed some decline until virtual microscopy became mainstream, topic that currently pushes research again.

Finally, the number of telepathology clinical trials is very low (1.9% of total publications). The higher but still low 4.7% figure for telemedicine papers [8] has been the reason for a call for more randomized studies [18], because the lack of clinical trials limits the application of evidence-based telemedicine. This is the case for telepathology (and related techniques) too: evidence-based telepathology will be the enabling factor for safely translating research into practice.

## Competing interests

The Author of the present paper is the third most prolific telepathology author, and there is some chance that with this paper may reach the first two.

## Authors' contributions

VDM designed the study, developed the software, analysed data and wrote the paper.

## Supplementary Material

Additional file 1 **XML file used for analysis** The enclosed telepathologyreferences.xml file contains all data related to the articles enclosed in the study. Click here for file
